# Identification of Hunnivirus in Bovine and Caprine Samples in North America

**DOI:** 10.3390/v17111491

**Published:** 2025-11-11

**Authors:** Suzanna Storms, Ailam Lim, Christian Savard, Yaindrys Rodriguez Olivera, Sandipty Kayastha, Leyi Wang

**Affiliations:** 1 Veterinary Diagnostic Laboratory, Department of Veterinary Clinical Medicine, College of Veterinary Medicine, University of Illinois, Urbana, IL 61802, USA; storms1@illinois.edu (S.S.); 2 Wisconsin Veterinary Diagnostic Laboratory, University of Wisconsin-Madison, Madison, WI 53711, USA; allim2@wisc.edu; 3 Biovet Inc., Saint-Hyacinthe, QC J2S 8W2, Canada; christian.savard@antechdx.com (C.S.)

**Keywords:** Picornaviridae, next-generation sequencing, bovine, caprine, diarrheal disease

## Abstract

Diarrhea in young ruminants is a global issue and causes significant economic losses worldwide. In addition to common pathogens like rotavirus, coronavirus, and astrovirus, new viruses can be identified through unbiased next-generation sequencing (NGS) techniques. Here, we report the initial identification of a hunnivirus from a one-month-old goat with diarrhea using shotgun metagenomic NGS. A complete hunnivirus genome was recovered. Phylogenetic tree analysis revealed that this goat hunnivirus was more closely related to cattle hunnivirus than to small ruminant hunnivirus strains, suggesting a prior cross-species transmission event. The genome was used to design primers/probes for the conserved 3D^pol^ RdRP gene for real-time RT-PCR to screen banked ruminant fecal samples. Screening of 144 ruminant fecal samples showed that 9 of 38 goat, 22 of 98 cattle, and 0 of 8 sheep samples were positive for hunnivirus. Sequencing of the 3D^po^ region was performed on selected positive samples and revealed two lineages of hunnivirus circulating in North America. Our study highlights the importance of further investigation and monitoring of fecal samples using unbiased metagenomic tools to identify potential pathogens or co-infections in ruminants.

## 1. Introduction

Hunniviruses are small, non-enveloped, single-stranded, positive-sense RNA viruses of the *Picornaviridae* family. Picornaviruses are genetically diverse with 68 genera. Picornaviruses usually have one, or a small group of, natural host species, and are typically transmitted by fecal–oral or airborne routes [1]. Picornaviruses cause diseases in humans and animals that range from subclinical, mild, to severe systemic diseases, with some examples being the common cold, diarrhea, poliomyelitis, hepatitis A, and foot-and-mouth disease [2]. Picornavirus virions are about 30–32 nm diameter in size and consist of 60 identical protomers comprising the capsid. Picornaviruses are resistant to ether, chloroform, and non-ionic detergents [1]. Hunniviruses share the general picornaviral characteristics but are about 28 nm in diameter, with an icosahedral capsid surrounding naked RNA of about 7.5 kb in length with a single ORF [3].

The hunnivirus genome is comprised of three regions, the P1, P2, and P3 regions. The P1 region codes for structural proteins, and P2 and P3 encode for non-structural proteins [4]. P3 contains the RNA-dependent RNA polymerase, also known as the 3D polymerase (3D^pol^), used in viral genome replication [5].

The *Hunnivirus* genus derives its name from the geographic locations Hungary and Northern Ireland, where the first three hunniviruses were originally identified. Currently, the *Hunnivirus* genus consists only one species: *Hunnivirus amagyari* (formerly *Hunnivirus* A) [6]. Hunnivirus was initially discovered in ovine cell cultures in 1965 in Northern Ireland, and additional strains were identified from clinically heathy cattle and sheep in Hungary in 2008–2009 [7]. Since then, more genotypes of hunnivirus have been reported in goats, water buffalo, pangolins, Norway rats, greater bandicoot rats, Asian house rats, and ricefield rats [4,7,8,9,10].

In the present study, we report the identification of hunnivirus in a diarrheal goat in Illinois, USA. Based on this finding, we hypothesized that hunnivirus may be present in several ruminant species in North America and retrospectively screened for the virus using banked samples from cattle, sheep, and goats to demonstrate the presence of the virus in North American ruminants. Few studies have explored the role of hunnivirus in animal disease; however, they do report that hunniviruses may contribute to diarrhea in cats, cattle, and sheep [11,12].

## 2. Materials and Methods

### 2.1. Study Design

Our first hunnivirus case was identified from a submission of a one-month-old diarrheic goat submitted to the University of Illinois Veterinary Diagnostic Lab. Whole-genome sequencing of feces from the goat was performed, and hunnivirus was identified (see metagenomic sequencing and analysis section). After identification, primers/probes were designed, and convenience samples of previously archived fecal samples were screened using RT-PCR assays to determine the presence of hunnivirus in feces of healthy and diseased ruminants in North America. This is a retrospective study of ruminant feces submitted to three diagnostic laboratories, two in the midwestern United States and one in Quebec, Canada.

### 2.2. Samples

Fecal samples from cattle (n = 98), sheep (n = 8), and goats (n = 38) submitted by veterinarians to the University of Illinois Veterinary Diagnostic Lab, University of Wisconsin Veterinary Diagnostic Lab, and Biovet Diagnostic lab (Quebec, Canada) were used for molecular testing in the present study. Samples were submitted for a diarrheal testing panel or other fecal testing between 2019 and 2024. Some of the small ruminant samples from Canada were submitted for intestinal parasite screens and not specifically for diarrhea. The time from collection to receipt by the lab was variable. Ancillary treatment data were not provided for the samples included in this study. Animals with species data included in this study can be found in Supplementary Table S1.

IACUC approval was not necessary for this study, in accordance with the NIH Office of Laboratory Animal Welfare, guidance NOT-OD-23-119, as the samples were obtained from privately owned animals receiving routine care, without the purpose of generating data for research purposes.

### 2.3. RNA Extraction

Fecal nucleic acid extractions took place at the University of Illinois Veterinary Diagnostic Lab and the Biovet Diagnostic lab. The US samples were processed at the University of Illinois Veterinary Diagnostic Lab, where fecal samples of different animals were swabbed and placed into 1.5 mL centrifuge tubes containing 0.5 mL of 1× PBS solution. The swabs were agitated in the solution and removed. The tubes were then briefly vortexed, followed by centrifugation for 2 min at 6800 rcf. The first extraction method used 100 µL of fecal supernatant for total nucleic acid extraction with the BioSprint 96 One-For-All Vet kit (Qiagen, Germantown, MD, USA).

Fecal samples from the Biovet Diagnostic lab were diluted in a 1:5 *w/v* ratio in a tube containing PBS with bashing beads for lysis and homogenization. One hundred microliters of supernatant was used for nucleic acid extraction using the Universal Pathogen DNA/RNA Extraction Kit (Galenvs Sciences, Montreal, QC, Canada).

### 2.4. Metagenomic Sequencing, Assembly, and Analysis

All sequencing was performed at the University of Illinois Veterinary Diagnostic Laboratory. Metagenomic sequencing of the goat feces from the index case was performed using a sequence-independent single-primer amplification (SISPA) protocol, and a sequencing library was prepared using the Nextera XT DNA Library Preparation Kit (Illumina, San Diego, CA, USA) as described previously [13]. The samples were then sequenced using the MiSeq Reagent Kits V2 (Illumina, San Diego, CA, USA) at 300 cycles on the MiSeq platform. De novo assembly of FASTQ files was performed in FASTA using SPAdes [14,15], and local blasts of contigs were run against the NCBI nt local database. Kraken was used to identify raw reads and provide visualization using Krona [16,17]. Sequence alignment and phylogenetic tree analysis were performed using MEGA software version 7.0.26, with 1000 replicates used for bootstrap analysis [18].

### 2.5. Real-Time RT-PCR Detection of Ruminant Hunnivirus and 3D^pol^ Gene Partial Sequencing

Primers and probes were designed based upon the metagenomic sequencing results for hunnivirus. Two forward and two reverse primers along with a probe targeting the RNA-dependent RNA polymerase (also known as 3D polymerase, 3D^pol^) region of the hunnivirus sequences were designed and manufactured by Integrated DNA Technologies (Coralville, IA, USA). The primers and probes used in the assay can be found in Table 1. Primer specificity was assessed using the online Primer-BLAST at NCBI, and the specificity results can be found in Supplementary Table S2.

Following verification of the primer and probe design, extracted nucleic acid samples from banked ruminant samples were subjected to real-time RT-PCR for hunnivirus screening. The TaqPath 1-Step Multiplex Master Mix (Applied Biosystems, Watham, MA, USA, cat. no. A28525) was used for the PCR assays. Briefly, for each reaction, 6.25 μL of 4X TaqPath 1-Step Master Mix, 1 μL of each primer (10 μM concentration), 1 μL of the probe (5 μM), 5 μL of RNA, and 8.75 μL of nuclease-free water were used, for a total volume of 25 μL per reaction. Real-time RT-PCR was run on the Bio-Rad CFX Opus 96 Real-Time thermocycler (Bio-Rad, Hercules, CA, USA) with the following conditions: UNG incubation at 25 °C for 2 min, reverse transcription at 48 °C for 10 min, PCR activation and reaction at 95 °C for 10 min, and 40 cycles of 95 °C for 15 s and 60 °C for 45 s Results were analyzed using the CFX Maestro software (Bio-Rad, Hercules, CA, USA, version 1.0).

Conventional RT-PCRs were performed using the SuperScript™ III One-Step RT-PCR System with Platinum™ Taq DNA Polymerase (Invitrogen, Carlsbad, CA, USA, cat. no. 12574026). Briefly, 12.5 μL of 2X Reaction Mix, 1 μL of forward primer (10 μM), 1 μL of reverse primer (10 μM), 1 μL of SuperScript™ III RT/Platinum™ Taq Mix, 5 μL of RNA template, and 4.5 μL of nuclease-free water were used for each 25 μL reaction. The RT-PCR was run using the following thermocycler conditions: reverse transcription at 45 °C for 45 min; PCR activation and reaction at 94 °C for 2 min, 40 cycles of 94 °C for 30 s, 50 °C for 30 s, and 68 °C for 1 min; and a final incubation at 68 °C for 5 min.

The conventional RT-PCR product was separated and visualized by electrophoresis, and bands were cut from the agarose gel and purified using the QIAquick Gel Extraction Kit (Qiagen, Germantown, MD, USA, cat. no. 28704). Purified nucleic acids were sequenced using the Sanger sequencing method to confirm the sequence identity. Sequences were deposited in the GenBank database.

### 2.6. Data Analysis

*Salmonella* spp. and bovine kobuvirus PCR data were available for 48 of the bovine samples. A chi-squared test of independence was performed to determine whether the various infection agents were related, with a null hypothesis of no association. OriginPro software (version 2023) was used to perform the analysis. Additionally, age data were available for 47 of the 96 samples. These data were subjected to Pearson, Spearman, and Kendall correlation calculations. Healthy and diarrheic bovine fecal samples were analyzed using a chi-squared test of independence to determine evidence of association between hunnivirus detection and diarrhea.

## 3. Results

### 3.1. Identification of Hunnivirus with Metagenomic Sequencing

A one-month-old goat with diarrhea was submitted for necropsy evaluation to the University of Illinois Veterinary Diagnostic Laboratory. Its fecal samples (IL43480) tested negative for rotavirus, bovine coronavirus, and parasites. Metagenomic sequencing of the feces indicated that 26% of the reads were classified as bovine hungarovirus 1 by Kraken analysis (Supplementary Figure S1). De novo assembly produced a 7400 nt contig, and a local BLAST of the files revealed that it was closely related to bovine hunnivirus strains previously identified.

**Figure 1:**
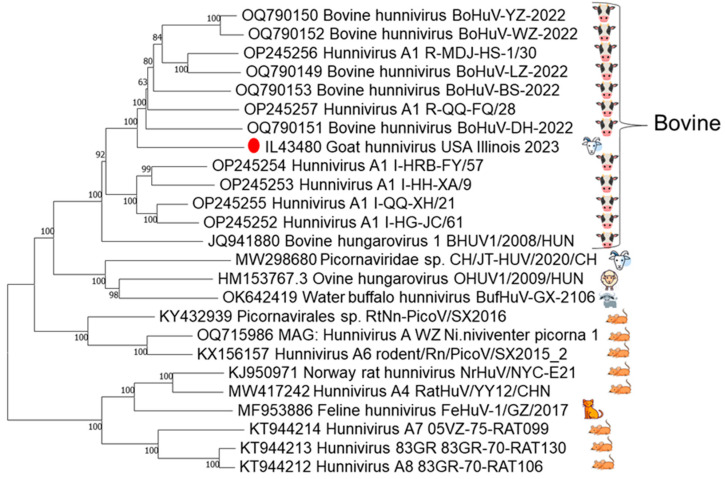
Index case phylogenetic analysis. Phylogenetic reconstruction of hunnivirus complete genomes retrieved from GenBank, with bootstrap support indicated at each node. Host species of virus are depicted on the right. The North American caprine index case, denoted by the red dot, demonstrates closer genomic affinity with bovine strains than with previously characterized caprine or ovine strains.

The final genome assembly for the full-genome sequence of this goat hunnivirus strain IL43480 was 7441 nt in length and deposited in GenBank. The GenBank accession number for the sequence is PV935296. BLASTn comparison of the goat hunnivirus strain IL43480 genome showed 83–84% identity to complete hunnivirus genomes, with the highest identity shared with the bovine hunnivirus strain BoHuV-YU-2002 (OQ790150.1) [19]. Phylogenetic analysis of hunnivirus genomes further confirmed that this strain clustered more closely with bovine strains than with published caprine, ovine, and water buffalo strains (Figure 1).

### 3.2. Real-Time RT-PCR Screening of Archived Samples

**Figure 2:**
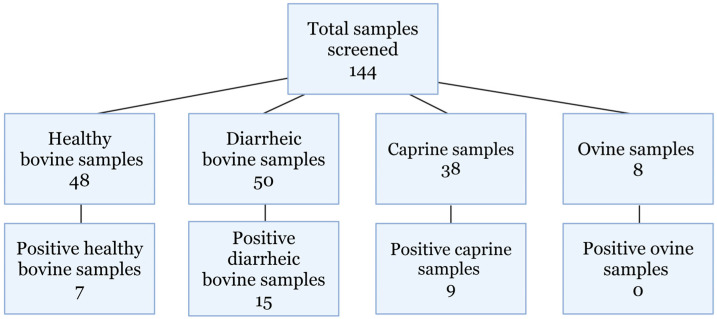
Samples screened. Total samples screened for hunnivirus detection using in-house designed RT-PCR primers.

Real-time RT-PCR screening of archived samples identified 31 positive samples (Figure 2). The samples originated from animals that were 5 days old to 10 years old. Ninety-eight bovine samples were screened, with 48 healthy and 50 diarrheic samples, of which there were 7 and 15 positive identifications, respectively. Thirty-eight caprine and eight ovine samples from both diarrheic and healthy animals were screened, and nine caprine samples were positive for hunnivirus. Additionally, 48 of the 50 bovine samples were previously screened for *Salmonella* spp. and bovine kobuvirus. Diarrheic bovine, caprine, and ovine hunnivirus Ct values are presented in Table 2, with healthy bovine sample information provided in Supplementary Table S1.

Forty-eight bovine samples submitted for a diarrhea panel were analyzed to determine the association between *Salmonella* ssp., bovine kobuvirus, and hunnivirus co-infection in samples. One sample was co-infected with *Salmonella*, hunnivirus, and bovine kobuvirus (sample 25, Table 2), and twelve were co-infected with bovine kobuvirus and hunnivirus. Seven were co-infected with *Salmonella* spp. and bovine kobuvirus.

Chi-square analysis showed no significant evidence of association between *Salmonella* spp. and hunnivirus co-infection (*p* = 0.067), and no significant evidence of association between *Salmonella* spp. and bovine kobuvirus detection (*p* = 0.60). However, there was significant evidence of association found between hunnivirus and bovine kobuvirus (*p* = 0.05), which can be interpreted as a negative association. The chi-square results can be seen in Supplementary Table S3.

We also compared the 48 diarrhea panel samples with 48 healthy bovine fecal samples using a chi-squared test. In total, 15 of the 48 diarrheic samples were positive for hunnivirus, while 7/48 healthy bovine samples were positive for hunnivirus. We found that there was no significant evidence of association between hunnivirus PCR-positive samples and diarrheic samples (*p* = 0.09) (Supplementary Table S3).

Normality of age and PCR detection data were assessed using the Shapiro–Wilk test, which indicated that both bovine and caprine samples were not normally distributed. Spearman and Kendall correlations were calculated between age and hunnivirus shedding, and no correlation was seen (Supplementary Table S4).

### 3.3. The 3D^pol^ Partial Genome Sequencing

**Figure 3:**
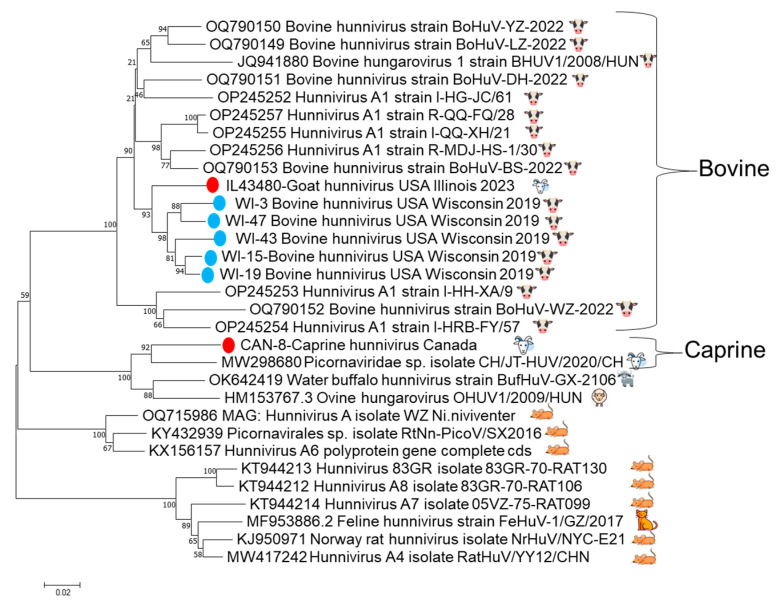
The trimmed 666 bp partial 3D^pol^ gene phylogenetic tree. Included in the tree are the six successfully sequenced 3D^pol^ amplicons from archived samples and the index case, with bootstrap support, indicated at each node. The five samples from cattle are shown as blue dots, and the Canadian goat and Illinois goat are shown with red dots. There is evidence of clustering of the red Illinois goat with the five blue sequences from Wisconsin, as well as other samples from bovine hosts, including the original bovine hungarovirus from 2008, separate from the linage that consists of the Canadian caprine strain and other previously sequenced sheep, goat, and water buffalo strains.

Conventional PCR primers were designed to produce an 803 nt amplicon for further characterization of positive samples. Seven hunnivirus real-time RT-PCR samples with a Ct value ≤ 30 were subsequently amplified with the conventional primers to produce a 3D^pol^ amplicon for sequencing. Six of the seven samples were successfully amplified and Sanger-sequenced. Phylogenetic tree analysis of the 3D^pol^ partial genome (666 bp) indicated a USA cluster comprised of five USA cattle strains and the USA caprine strain from this study inside a bovine lineage. One Canadian caprine strain clustered with another caprine strain from China within the sheep and goat lineage (Figure 3). The partial 3D^pol^ gene sequences are deposited in GenBank under the following accession numbers: PV94829-PV945834.

## 4. Discussion

Picornaviruses can cause significant neurological, cardiovascular, hepatic, respiratory, enteric, and vesicular diseases in various hosts. Although a related virus was isolated six decades ago, hunnivirus was genetically characterized in 2012 and officially designated in 2013 [20]. The clinical significance of hunniviruses in animals remains to be determined, since enteric illness has only been described in a few animals such as cattle, sheep, and cats. In the present study, we report the detection of a hunnivirus in a diarrheal goat, providing support for the idea that hunnivirus could be linked to diarrheal disease.

The heterogenous nature of the 3D polymerase gene of picornaviruses allows the design of viral species-specific primers for detection by PCR [5]. The hunnivirus sequenced from the index case allowed primers for the 3D^pol^ to be designed and subsequently detected hunnivirus in 24 additional samples that originated from cattle and goats. Analysis of partial 3D^pol^ sequences revealed two distinct geographic and host-specific lineages present in North America. This finding suggests that the Illinois goat case is most likely the result of a prior cross-species transmission event. The exact timing and route of the viral transmission from cattle to goats and the length of time that the bovine lineage has been circulating in goats warrant further investigation.

The presence of hunnivirus in diarrheic samples and healthy bovine fecal samples suggests that the virus could contribute to diarrhea and gastrointestinal disease in ruminants, but it is not the sole cause of gastrointestinal disease, such that it warrants further surveillance and investigation. The ovine and caprine samples used in this study were not specifically from diarrhea panels and comprised a lower percentage of positive samples compared to the diarrheic cattle samples (19.5% positive for goats and sheep vs. 30% positive for diarrheic cattle vs. 14.5% for healthy cattle). With the limited data available, no correlation was found between age and hunnivirus detection in all species. The bovine samples covered both young calves and older animals. Ovine and caprine samples were largely limited to animals over one year of age, with the exception of the index case. Additionally, few ovine fecal samples were available, as limited submissions for parasitology testing resulted in fewer banked specimens, representing another limitation of this study.

The interpretation of our findings is limited due to several factors, primarily the nature of the samples used in the study. The samples used were convenience samples banked by diagnostic laboratories. The clinical history for each submission is unknown. The authors had no control over the sample handling (collection, storage, and transport) prior to reception at the laboratories. In addition, adjunct therapies and metadata surrounding the samples were only as complete as the submission forms that accompanied the samples. The cattle samples were from diarrhea panels, which could potentially have increased the likelihood of our finding evidence of the virus, and the amount of hunnivirus in healthy cattle is unknown due to the lack of screened samples. Additionally, due to the low number of ovine samples in our study, we are unable to describe the point prevalence in this species.

## 5. Conclusions

Our findings expand the geographic range of hunnivirus and suggest the existence of multiple viral lineages in North American ruminants. This study reinforces the need for further investigation into the role hunnivirus plays in gastrointestinal illness in these species. Future studies investigating the demographics associated with a higher risk for infection or disease associated with hunnivirus would be a viable next step. The results reported here demonstrate that unbiased metagenomic sequencing has a place in veterinary diagnostic investigations and can contribute to the discovery and surveillance of emerging viruses in the veterinary field.

## Figures and Tables

**Table 1:** Primers and probes used in the conventional and real-time RT-PCR. For complete information regarding T_m_ and self-complementarity, see Supplementary Table S2. Degenerate code in primers used in the conventional PCR: R: A or G; W: A or T; D: A or G or T.

PCR Type	Primer Name	Oligonucleotide Sequence (5′ > 3′)	Amplicon
Real-time RT-PCR	Rum-HunniV-For-1	CAGGTGAATGAACGACTGTC	189 nt
	Rum-HunniV-For-2	CCTGTGAATGAACGGCTGTC	
	Rum-HunniV-Rev-1	CTTCCATCATAAGCATCAGTTC	
	Rum-HunniV-Rev-2	CTTCCATCATGAGCATCAGTTC	
	Rum-HunniV-Probe	FAM/TGCACGAGG/ZEN/CAGTCTTTGGAAC/IABkFQ	
Conventional PCR	HunniV-Pan-For	GAGGCAGTCTTTGGRACWGACAA	803 nt
	HunniV-Pan-Rev-2	CCACTCTTTGAAGCTGGDGTAAT	

**Table 2:** Sample information for diarrheic bovine samples and all caprine and ovine samples included in the study, including animal location; PCR results for hunnivirus, *Salmonella* sp., and bovine kobuvirus (Ct values); and age in weeks. Healthy bovine sample information can be found in Supplementary Table S1.

Animal ID	Species	Animal Location	Hunnivirus Ct	*Salmonella* spp. Ct	Bovine Kobuvirus Ct	Age in Weeks *
1	*Bovine*	IL	31.80	ND	ND	Unknown
2	*Bovine*	WI	0.00	36.80	20.58	1
3	*Bovine*	WI	0.00	ND	ND	Unknown
4	*Bovine*	WI	28.56	0.00	19.48	1
5	*Bovine*	WI	0.00	0.00	32.14	16
6	*Bovine*	OH	0.00	0.00	0.00	16
7	*Bovine*	WI	0.00	0.00	0.00	52 *
8	*Bovine*	WI	0.00	39.00	0.00	<1
9	*Bovine*	WI	0.00	0.00	0.00	104 *
10	*Bovine*	WI	0.00	0.00	0.00	104 *
11	*Bovine*	OH	35.73	0.00	0.00	5
12	*Bovine*	WI	30.10	0.00	28.00	32 *
13	*Bovine*	WI	0.00	0.00	19.93	3
14	*Bovine*	WI	0.00	0.00	22.37	2
15	*Bovine*	WI	29.76	0.00	20.38	1
16	*Bovine*	WI	38.24	0.00	23.10	1
17	*Bovine*	WI	37.35	0.00	22.00	1
18	*Bovine*	WI	38.65	0.00	18.90	1
19	*Bovine*	WI	25.36	0.00	24.55	1
20	*Bovine*	NM	0.00	33.40	25.29	1
21	*Bovine*	NM	0.00	34.00	18.22	1
22	*Bovine*	NM	0.00	31.90	19.28	1
23	*Bovine*	NM	0.00	0.00	18.68	1
24	*Bovine*	NM	0.00	36.90	19.29	1
25	*Bovine*	NM	32.29	37.10	19.67	1
26	*Bovine*	WI	38.01	0.00	20.06	Unknown
27	*Bovine*	WI	0.00	0.00	21.46	1
28	*Bovine*	WI	0.00	26.50	0.00	<1
29	*Bovine*	OH	33.09	0.00	36.10	10
30	*Bovine*	WI	0.00	0.00	24.98	1
31	*Bovine*	WI	0.00	0.00	29.23	Unknown
32	*Bovine*	WI	0.00	0.00	39.12	Unknown
33	*Bovine*	WI	0.00	29.10	0.00	208 *
34	*Bovine*	WI	0.00	39.00	0.00	208 *
35	*Bovine*	WI	0.00	0.00	0.00	Unknown
36	*Bovine*	WI	0.00	0.00	38.46	Uunknown
37	*Bovine*	WI	0.00	0.00	0.00	Unknown
38	*Bovine*	WI	0.00	0.00	0.00	Unknown
39	*Bovine*	WI	0.00	0.00	39.34	468 *
40	*Bovine*	WI	0.00	0.00	0.00	Unknown
41	*Bovine*	WI	0.00	24.00	37.77	156 *
42	*Bovine*	WI	0.00	0.00	39.75	Unknown
43	*Bovine*	WI	29.77	0.00	24.01	3
44	*Bovine*	WI	0.00	0.00	0.00	Unknown
45	*Bovine*	WI	0.00	30.30	0.00	Unknown
46	*Bovine*	OH	0.00	0.00	22.15	2
47	*Bovine*	WI	27.18	0.00	0.00	Unknown
48	*Bovine*	WI	0.00	0.00	0.00	Unknown
49	*Bovine*	MI	30.41	0.00	20.10	1
50	*Bovine*	WI	0.00	0.00	16.13	1
51	*Caprine*	IL	0.00	ND	ND	4
52	*Caprine*	CAN	0.00	ND	ND	104 *
53	*Caprine*	CAN	0.00	ND	ND	104 *
54	*Caprine*	CAN	0.00	ND	ND	104 *
55	*Caprine*	CAN	36.31	ND	ND	8 *
56	*Caprine*	CAN	23.85	ND	ND	8 *
57	*Caprine*	CAN	33.37	ND	ND	8 *
58	*Caprine*	CAN	0.00	ND	ND	52 *
59	*Caprine*	CAN	0.00	ND	ND	52 *
60	*Caprine*	CAN	0.00	ND	ND	52 *
61	*Caprine*	CAN	36.37	ND	ND	52 *
62	*Caprine*	CAN	0.00	ND	ND	52 *
63	*Caprine*	CAN	35.99	ND	ND	52 *
64	*Caprine*	CAN	0.00	ND	ND	104 *
65	*Caprine*	CAN	0.00	ND	ND	104 *
66	*Caprine*	CAN	0.00	ND	ND	104 *
67	*Caprine*	CAN	0.00	ND	ND	104 *
68	*Caprine*	CAN	0.00	ND	ND	104 *
69	*Caprine*	CAN	37.63	ND	ND	104 *
70	*Caprine*	CAN	0.00	ND	ND	104 *
71	*Caprine*	CAN	0.00	ND	ND	104 *
72	*Caprine*	CAN	0.00	ND	ND	104 *
73	*Caprine*	CAN	0.00	ND	ND	104 *
74	*Caprine*	WI	0.00	ND	ND	52 *
75	*Caprine*	WI	0.00	ND	ND	104 *
76	*Caprine*	WI	0.00	ND	ND	104 *
77	*Caprine*	WI	39.35	ND	ND	156 *
78	*Caprine*	WI	38.46	ND	ND	Unknown
79	*Caprine*	WI	0.00	ND	ND	Unknown
80	*Caprine*	WI	0.00	ND	ND	104 *
81	*Caprine*	WI	0.00	ND	ND	260 *
82	*Caprine*	WI	0.00	ND	ND	520 *
83	*Caprine*	WI	0.00	ND	ND	260 *
84	*Caprine*	WI	0.00	ND	ND	156 *
85	*Caprine*	WI	35.26	ND	ND	104 *
86	*Caprine*	WI	0.00	ND	ND	104 *
87	*Caprine*	WI	0.00	ND	ND	Unknown
88	*Caprine*	WI	0.00	ND	ND	Unknown
89	*Ovine*	IL	0.00	ND	ND	Unknown
90	*Ovine*	WI	0.00	ND	ND	208 *
91	*Ovine*	WI	0.00	ND	ND	Unknown
92	*Ovine*	CAN	0.00	ND	ND	104 *
93	*Ovine*	CAN	0.00	ND	ND	104 *
94	*Ovine*	CAN	0.00	ND	ND	104 *
95	*Ovine*	CAN	0.00	ND	ND	104 *
96	*Ovine*	CAN	0.00	ND	ND	104 *

ND—indicates missing data where PCR was not performed. 0.00—indicates no nucleic acid detection when PCR was performed. * Age is rounded down to the nearest reported week. For example, a one-year-old animal is reported here as 52 weeks, and an animal less than 7 days old is reported as <1 week.

## Data Availability

All data related to the current study are provided in the manuscript, and sequences were deposited in GenBank, with accession numbers provided in the text.

## Supplementary Materials

The following supporting information can be downloaded at https://www.mdpi.com/article/10.3390/v17111491/s1, Supplementary Table S1. Complete animal data.; Supplementary Table S2. Primer pair specificity, Tm, and ancillary information; Supplementary Table S3: Contingiency tables and Chi-Square tests between the presence of hunnivirus, Salmonella, and bovine kobuvirus; Supplementary Table S4. Correlation coefficients between host age and hunnivirus detection, represented as a binomial. Supplementary Figure S1. Kraken Taxonomical Classification Analysis of raw FASTQ files for the caprine index case feces IL43480 showing 26% of reads classified as related to bovine hungarovirus 1 (in yellow).
