# Correction: Storms et al. Identification of Hunnivirus in Bovine and Caprine Samples in North America. *Viruses* 2025, *17*, 1491

**DOI:** 10.3390/v18010106

**Published:** 2026-01-13

**Authors:** Suzanna Storms, Ailam Lim, Christian Savard, Yaindrys Rodriguez Olivera, Sandipty Kayastha, Leyi Wang

**Affiliations:** 1 Veterinary Diagnostic Laboratory, Department of Veterinary Clinical Medicine, College of Veterinary Medicine, University of Illinois, Urbana, IL 61802, USA; storms1@illinois.edu (S.S.);; 2 Wisconsin Veterinary Diagnostic Laboratory, University of Wisconsin-Madison, Madison, WI 53711, USA; allim2@wisc.edu; 3 Biovet Inc., Saint-Hyacinthe, QC J2S 8W2, Canada; christian.savard@antechdx.com (C.S.);

**Text Correction**

There were errors in the original publication [1]. The total number of healthy cattle was incorrect and the total number of cattle screened (sum of healthy and diarrheic cattle) was also incorrect. The corrected numbers are as follows:

Abstract

“Screening of 144 ruminant fecal samples showed that 9 of 38 goat, 22 of 98 cattle, and 0 of 8 sheep samples were positive for hunnivirus.”

*Section 2.2. Samples,*

“Fecal samples from cattle (n = 98)”

*3.2. Real-Time RT-PCR Screening of Archived Samples*

“Real-time RT-PCR screening of archived samples identified 31 positive samples (Figure 2). The samples originated from animals that were 5 days old to 10 years old. Ninety-eight bovine samples were screened, with 48 healthy and 50 diarrheic samples, of which there were 7 and 15 positive identifications, respectively. Thirty-eight caprine and eight ovine samples from both diarrheic and healthy animals were screened, and nine caprine samples were positive for hunnivirus. Additionally, 48 of the 50 bovine samples were previously screened for *Salmonella* spp. and bovine kobuvirus. Diarrheic bovine, caprine, and ovine hunnivirus Ct values are presented in Table 2, with healthy bovine sample information provided in Supplementary Table S1”.

**Error in Figure 2**

In the original publication [1], there was a mistake in Figure 2. Samples screened. The total number of samples screened was incorrect. The correct figure is below.

**Figure 2:**
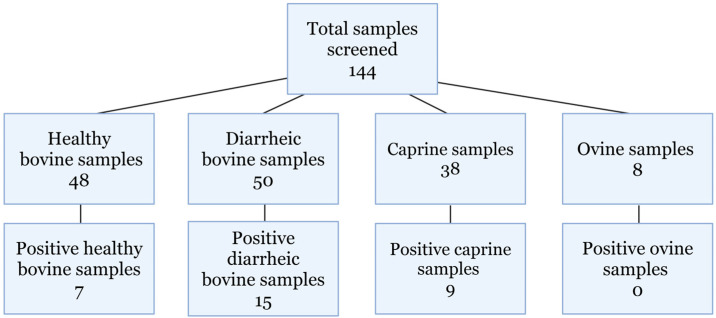
Samples screened. Total samples screened for hunnivirus detection using in-house designed RT-PCR primers.

In the original publication [1], there was a mistake in Supplementary Table S1. Two extra-healthy bovine samples were incorrectly included. The correct table is below.

The authors state that the scientific conclusions are unaffected. This correction was approved by the Academic Editor. The original publication has also been updated.

## Figures and Tables

**Table S1:** Complete animal data. **Healthy bovine fecal samples**.

Animal ID	Hunnivirus PCR Ct
NTC	0.00
PAC	24.25
NTC	0.00
PAC	25.26
WI-102	0.00
WI-103	0.00
WI-105	0.00
WI-110	0.00
WI-120	0.00
WI-124	0.00
WI-125	0.00
WI-127	34.50
WI-131	0.00
WI-132	34.38
WI-134	34.55
WI-135	0.00
WI-144	0.00
WI-156	0.00
WI-157	0.00
WI-158	0.00
WI-159	0.00
WI-161	0.00
WI-171	36.62
WI-177	0.00
WI-178	0.00
WI-182	0.00
WI-183	0.00
WI-184	0.00
WI-185	0.00
WI-192	0.00
WI-193	33.66
WI-194	0.00
WI-196	0.00
WI-198	0.00
WI-199	0.00
WI-205	0.00
WI-212	0.00
WI-216	37.46
WI-232	0.00
WI-234	0.00
WI-236	0.00
WI-246	0.00
WI-258	0.00
WI-263	0.00
WI-265	0.00
WI-267	0.00
WI-268	0.00
WI-274	27.13
WI-276	0.00
WI-278	0.00
WI-280	0.00
WI-281	0.00
