# Supplementary material for: Identification of Hunnivirus in Bovine and Caprine Samples in North America
Source: Viruses. 2025 Nov 11;17(11):1491. doi: 10.3390/v17111491 (PMC12656811; doi:10.3390/v17111491)
Supplement: Supplementary file 1 [file viruses-17-01491-s001.zip › viruses-3911453-supplementary-1.pdf]

Supplementary Table S1. Complete animal data.

Tube ID	Species	Hunnivirus Ct	Salmonella Ct	BKV Ct	age in weeks	Location
24-31535	Bovine	31.80				IL
Wisconsin-01	Bovine	0.00	36.8	20.58	1	WI
WI-RNA-03	Bovine	0.00				WI
Wisconsin-03	Bovine	28.56	0	19.48	1	WI
Wisconsin-05	Bovine	0.00	0	32.14	16	WI
Wisconsin-06	Bovine	0.00	0	0	16	OH
Wisconsin-07	Bovine	0.00	0.00	0.00	52	WI
Wisconsin-08	Bovine	0.00	39.00	0.00	0	WI
Wisconsin-09	Bovine	0.00	0.00	0.00	104	WI
Wisconsin-10	Bovine	0.00	0.00	0.00	104	WI
WI-RNA-11	Bovine	35.73	0.00	0.00	5	OH
WI-RNA-12	Bovine	30.10	0.00	28.00	32	WI
Wisconsin-13	Bovine	0.00	0.00	19.93	3	WI
Wisconsin-14	Bovine	0.00	0.00	22.37	2	WI
WI-RNA-15	Bovine	29.76	0.00	20.38	1	WI
WI-RNA-16	Bovine	38.24	0.00	23.10	1	WI
WI-RNA-17	Bovine	37.35	0.00	22.00	1	WI
WI-RNA-18	Bovine	38.65	0.00	18.90	1	WI
WI-RNA-19	Bovine	25.36	0.00	24.55	1	WI
Wisconsin-20	Bovine	0.00	33.40	25.29	1	NM
Wisconsin-21	Bovine	0.00	34.00	18.22	1	NM
Wisconsin-22	Bovine	0.00	31.90	19.28	1	NM
Wisconsin-23	Bovine	0.00	0.00	18.68	1	NM
Wisconsin-24	Bovine	0.00	36.90	19.29	1	NM
WI-RNA-25	Bovine	32.29	37.10	19.67	1	NM
WI-RNA-26	Bovine	38.01	0.00	20.06		WI
Wisconsin-27	Bovine	0.00	0.00	21.46	1	WI
Wisconsin-28	Bovine	0.00	26.50	0.00	0	WI
WI-RNA-29	Bovine	33.09	0.00	36.10	10	OH
Wisconsin-30	Bovine	0.00	0.00	24.98	1	WI
Wisconsin-31	Bovine	0.00	0.00	29.23		WI
Wisconsin-32	Bovine	0.00	0.00	39.12		WI
Wisconsin-33	Bovine	0.00	29.10	0.00	208	WI
Wisconsin-34	Bovine	0.00	39.00	0.00	208	WI
Wisconsin-35	Bovine	0.00	0.00	0.00		WI
Wisconsin-36	Bovine	0.00	0.00	38.46		Sal
Wisconsin-37	Bovine	0.00	0.00	0.00		WI
Wisconsin-38	Bovine	0.00	0.00	0.00		WI
Wisconsin-39	Bovine	0.00	0.00	39.34	468	WI
Wisconsin-40	Bovine	0.00	0.00	0.00		WI
Wisconsin-41	Bovine	0.00	24.00	37.77	156	WI
Wisconsin-42	Bovine	0.00	0.00	39.75		WI
WI-RNA-43	Bovine	29.77	0.00	24.01	3	WI
Wisconsin-44	Bovine	0.00	0.00	0.00		WI
Wisconsin-45	Bovine	0.00	30.30	0.00		WI
Wisconsin-46	Bovine	0.00	0.00	22.15	2	OH
WI-RNA-47	Bovine	27.18	0.00	0.00		WI
Wisconsin-48	Bovine	0.00	0.00	0.00		WI
WI-RNA-49	Bovine	30.41	0.00	20.10	1	MI
Wisconsin-50	Bovine	0.00	0.00	16.13	1	WI
21-40824	caprine	0.00				IL
1379142-1	Caprine	0.00				CAN
1379142-3	Caprine	0.00				CAN

1379142-5	Caprine	0.00		CAN
1379142-7	Caprine	36.31		CAN
1379142-8	Caprine	23.85		CAN
1379142-9	Caprine	33.37		CAN
1381743-1	Caprine	0.00		CAN
1381743-2	Caprine	0.00		CAN
1381743-3	Caprine	0.00		CAN
1381743-4	Caprine	36.37		CAN
1381743-5	Caprine	0.00		CAN
1381743-6	Caprine	35.99		CAN
1382176-1	Caprine	0.00		CAN
1382176-10	Caprine	0.00		CAN
1382176-11	Caprine	0.00		CAN
1382176-12	Caprine	0.00		CAN
1382176-4	Caprine	0.00		CAN
1382176-5	Caprine	37.63		CAN
1382176-6	Caprine	0.00		CAN
1382176-7	Caprine	0.00		CAN
1382176-8	Caprine	0.00		CAN
1382176-9	Caprine	0.00		CAN
WI-Feces-1	Caprine	0.00	52	WI
WI-Feces-11	Caprine	0.00	104	WI
WI-Feces-12	Caprine	0.00	104	WI
WI-Feces-14	Caprine	39.35	156	WI
WI-Feces-3	Caprine	38.46		WI
WI-Feces-4	Caprine	0.00		WI
WI-Feces-47	Caprine	0.00	104	WI
WI-Feces-49	Caprine	0.00	260	WI
WI-Feces5	Caprine	0.00	520	WI
WI-Feces-51	Caprine	0.00	260	WI
WI-Feces-52	Caprine	0.00	156	WI
WI-Feces-53	Caprine	35.26	104	WI
WI-Feces-8	Caprine	0.00	104	WI
WI-RNA-2	caprine	0.00		WI
WI-RNA-4	caprine	0.00		WI
21-40680	ovine	0.00		IL
WI-Feces-48	Ovine	0.00	208	WI
WI-Feces-50	Ovine	0.00		WI
1379142-16	ovine	0.00		WI
1379142-17	ovine	0.00		WI
1379142-18	ovine	0.00		WI
1379142-19	ovine	0.00		WI
1379142-24	ovine	0.00		WI

indicates no nucleic acids detected via PCR.

* empty cells are missing data points.

** age is rounded down to the nearest known week. Animals reported as one year were rounded down to 52 weeks.

Healthy bovine fecal samples.

Animal ID	Hunnivirus PCR Ct
NTC	0.00
PAC	24.25
NTC	0.00
PAC	25.26

WI-102	0.00
WI-103	0.00
WI-105	0.00
WI-110	0.00
WI-120	0.00
WI-124	0.00
WI-125	0.00
WI-127	34.50
WI-131	0.00
WI-132	34.38
WI-134	34.55
WI-135	0.00
WI-144	0.00
WI-156	0.00
WI-157	0.00
WI-158	0.00
WI-159	0.00
WI-161	0.00
WI-171	36.62
WI-177	0.00
WI-178	0.00
WI-182	0.00
WI-183	0.00
WI-184	0.00
WI-185	0.00
WI-192	0.00
WI-193	33.66
WI-194	0.00
WI-196	0.00
WI-198	0.00
WI-199	0.00
WI-205	0.00
WI-212	0.00
WI-216	37.46
WI-232	0.00
WI-234	0.00
WI-236	0.00
WI-246	0.00
WI-258	0.00
WI-263	0.00
WI-265	0.00
WI-267	0.00
WI-268	0.00
WI-274	27.13
WI-276	0.00
WI-278	0.00
WI-280	0.00
WI-281	0.00

Supplementary Table S2. Primer pair specificity, Tm, and ancillary information.

Forward Primer						
Primer pair name	Sequence (5'->3')	Length	Tm	GC%	Self complementarity	Self 3' complementarity
For-1, Rev-1	CAGGTGAATGAACGACTGTC	20	56.48	50	5	5
For-2, Rev-2	CCTGTGAATGAACGGCTGTC	20	59.2	55	4	2
Conventional PCR	GAGGCAGTCTTTGGNACNGACAA	23	56.57	52.38	4	2

Reverse Primer					
Sequence (5'->3')	Length	Tm	GC%	Self complementarity	Self 3' complementarity
CTTCCATCATAAGCATCAGTTC	22	54.97	40.91	3	0
CTTCCATCATGAGCATCAGTTC	22	57.18	45.45	6	1
CCACTCTTTGAAGCTGGNGTAAT	23	56.05	45.45	4	2

Accession numbers of specificity search.

NC_018668.1 Bovine hungarovirus 1 strain BHUV1/2008/HUN, complete genome.

NC_018668.1 Bovine hungarovirus 1 strain BHUV1/2008/HUN, complete genome.

NC_018668.1 Bovine hungarovirus 1 strain BHUV1/2008/HUN, complete genome,.

NC_025675.1 Norway rat hunnivirus isolate NrHuV/NYC-E21 polyprotein gene, complete cds.

Supplementary Table S3. Contingiency tables and Chi-Square tests between the presence of hunnivirus, Salmonella, and bovine kobuvirus. Hunnivirus and Salmonella contingency table and Chi-Square test

			Hunnivirus		
			0	1	Total
Salmonella	0	Count	23	13	36
	0	Expected Count	25.5	10.5	36
	0	Row%	63.88889	36.11111	100
	0	Col%	67.64706	92.85714	75
	0	Total%	47.91667	27.08333	75
	0	Residual	-2.5	2.5	
	0	Std.Residual	-0.49507	0.77152	
	0	Adj.Residual	-1.8334	1.8334	
	1	Count	11	1	12
	1	Expected Count	8.5	3.5	12
	1	Row%	91.66667	8.33333	100
	1	Col%	32.35294	7.14286	25
	1	Total%	22.91667	2.08333	25
	1	Residual	2.5	-2.5	
	1	Std.Residual	0.85749	-1.33631	
	1	Adj.Residual	1.8334	-1.8334	
	Total	Count	34	14	48
	Total	Expected Count	34	14	48
	Total	Row%	70.83333	29.16667	100
	Total	Col%	100	100	100
	Total	Total%	70.83333	29.16667	100

	ChiSquare	DF	Prob > ChiSq
Pearson Chi-Square	3.36134	1	0.06674
Likelihood Ratio	3.97317	1	0.04623
Linear Association	3.29132	1	0.06965
Continuity Correction	2.15126	1	0.14245

According to the Pearson Chi-Square test: At the 0.05 level, there is NOT significant evidence of association between two variables.

Hunnivirus and bovine kobuvirus contingiency table and Chi-Square test.

			Hunnivirus		
			0	1	Total
Bovine kobuvirus	0	Count	15	2	17
	0	Expected Count	12.04167	4.95833	17
	0	Row%	88.23529	11.76471	100
	0	Col%	44.11765	14.28571	35.41667
	0	Total%	31.25	4.16667	35.41667
	0	Residual	2.95833	-2.95833	
	0	Std.Residual	0.85252	-1.32855	
	0	Adj.Residual	1.96427	-1.96427	
	1	Count	19	12	31
	1	Expected Count	21.95833	9.04167	31
	1	Row%	61.29032	38.70968	100
	1	Col%	55.88235	85.71429	64.58333
	1	Total%	39.58333	25	64.58333
	1	Residual	-2.95833	2.95833	
	1	Std.Residual	-0.63132	0.98384	
	1	Adj.Residual	-1.96427	1.96427	
	Total	Count	34	14	48
	Total	Expected Count	34	14	48
	Total	Row%	70.83333	29.16667	100
	Total	Col%	100	100	100
	Total	Total%	70.83333	29.16667	100

	ChiSquare	DF	Prob > ChiSq
Pearson Chi-Square	3.85834	1	0.0495
Likelihood Ratio	4.25325	1	0.03918
Linear Association	3.77796	1	0.05193
Continuity Correction	2.66433	1	0.10262

According to the Pearson Chi-Square test: At the 0.05 level, there is significant evidence of association between two variables.

Salmonella and bovine kobuvirus contingiency table and Chi-Square test

			Bovine kobuvirus		
			0	1	Total
Salmonella	0	Count	12	24	36
	0	Expected Count	12.75	23.25	36
	0	Row%	33.33333	66.66667	100
	0	Col%	70.58824	77.41935	75
	0	Total%	25	50	75
	0	Residual	-0.75	0.75	
	0	Std.Residual	-0.21004	0.15554	
	0	Adj.Residual	-0.52273	0.52273	
	1	Count	5	7	12

	1	Expected Count	4.25	7.75	12
	1	Row%	41.66667	58.33333	100
	1	Col%	29.41176	22.58065	25
	1	Total%	10.41667	14.58333	25
	1	Residual	0.75	-0.75	
	1	Std.Residual	0.3638	-0.26941	
	1	Adj.Residual	0.52273	-0.52273	
	Total	Count	17	31	48
	Total	Expected Count	17	31	48
	Total	Row%	35.41667	64.58333	100
	Total	Col%	100	100	100
	Total	Total%	35.41667	64.58333	100

	ChiSquare	DF	Prob > ChiSq
Pearson Chi-Square	0.27324	1	0.60116
Likelihood Ratio	0.26918	1	0.60388
Linear Association	0.26755	1	0.60498
Continuity Correction	0.03036	1	0.86167

According to the Pearson Chi-Square test: At the 0.05 level, there is NOT significant evidence of association between two variables.

Bovine fecal samples from healthy and diarrheic animals contingency table and Chi-squared analysis.

			PCR negative	PCR positive	binomial
			1	0	Total
Diarrhea samples	0	Count	15	33	48
	0	Expected Count	11.5	36.5	48
	0	Row%	31.25	68.75	100
	0	Col%	65.21739	45.20548	50
	0	Total%	15.625	34.375	50
	0	Residual	3.5	-3.5	
	0	Std.Residual	1.03209	-0.57932	
	0	Adj.Residual	1.67382	-1.67382	
Healthy samples	1	Count	8	40	48
	1	Expected Count	11.5	36.5	48
	1	Row%	16.66667	83.33333	100
	1	Col%	34.78261	54.79452	50
	1	Total%	8.33333	41.66667	50
	1	Residual	-3.5	3.5	
	1	Std.Residual	-1.03209	0.57932	
	1	Adj.Residual	-1.67382	1.67382	
	Total	Count	23	73	96
	Total	Expected Count	23	73	96
	Total	Row%	23.95833	76.04167	100
	Total	Col%	100	100	100
	Total	Total%	23.95833	76.04167	100

	ChiSquare	DF	Prob > ChiSq
Pearson Chi-Square	2.80167	1	0.09417
Likelihood Ratio	2.83687	1	0.09212
Continuity Correction	2.05837	1	0.15137

According to the Pearson Chi-Square test: At the 0.05 level, there is NOT significant evidence of association between two variables.

Supplementary Table S4. Correlation coefficients between host age and hunnivirus detection, represented as a binomial.

	N	Mean	SD	Sum	Min	Max
binom	47.00	0.32	0.47	15.00	0.00	1.00
age	47.00	73.15	118.69	3438.00	0.00	520.00

	DF	Statistic	p-value	Decision at level(5%)
binom	47.00	0.59	<0.0001	Reject normality
age	47.00	0.67	<0.0001	Reject normality

Shaprio-Wilk test: at the 0.05 level, the data was not signficantly drwan from a normally distributed population.

		binom	age
binom	Spearman Corr.	1	-0.23303
binom	p-value	--	0.11494
age	Spearman Corr.	-0.23303	1
age	p-value	0.11494	--

		binom	age
binom	Kendall Corr.	1	-0.20337
binom	p-value	--	0.11399
age	Kendall Corr.	-0.20337	1
age	p-value	0.11399	--

The two correlation coefficients fail to reject the null hypothesis that age is correlated with hunnivirus detection in bovines.

	DF	Statistic	p-value	Decision at level(5%)
BINOM	38	0.53	<0.0001	Reject normality
AGE	34	0.66	<0.0001	Reject normality

Shaprio-Wilk test: at the 0.05 level, the data was not signficantly drwan from a normally distributed population.

		BINOM	AGE
BINOM	Spearman Corr.	1	-0.33495
BINOM	p-value	--	0.05282
AGE	Spearman Corr.	-0.33495	1
AGE	p-value	0.05282	--

		BINOM	AGE
BINOM	Kendall Corr.	1	-0.31
BINOM	p-value	--	0.07
AGE	Kendall Corr.	-0.31	1
AGE	p-value	0.07	--

The three correlation coefficients fail to reject the null hypothesis that age is correlated with hunnivirus detection in caprines.
