# Supplementary material for: Identification of Hunnivirus in Bovine and Caprine Samples in North America
Source: Viruses. 2025 Nov 11;17(11):1491. doi: 10.3390/v17111491 (PMC12656811; doi:10.3390/v17111491)
Supplement: Supplementary file 1 [file viruses-17-01491-s001.zip › viruses-3911453-Supplementary-Figure.pdf]

Supplementary materials-hunnivirus in bovine and caprine samples

Extraction protocols

Qiagen BioSprint 96 One-For-All Vet kit

RNA extraction was conducted using the Qiagen One-For-All kit following the manufacturer's instructions. For each sample, 40 µL of proteinase K was dispensed into the bottom of the sample well, followed by 200 µL of sample. Separately, a lysis mixture was prepared by combining 200 µL of Buffer AL, 200 µL of isopropanol, 25 µL of MagAttract Suspension G, and 2.7 µL of carrier RNA (1 µg/µL). This mixture was vortexed for 30 seconds, and then 400 µL was added to the corresponding sample well. Elution buffer (74–200 µL) was dispensed into the elution plate as needed. For the washing steps, four separate wash buffers were prepared: 700 µL of Buffer AW1 for Wash 1, 500 µL of Buffer AW1 for Wash 2, 500 µL of Buffer RPE for Wash 3, and 500 µL of Buffer RPE for Wash 4. All plates were loaded onto the BioSprint 96 system, and the extraction protocol was initiated by selecting the appropriate software program and following the plate loading prompts.

Applied Biosystems MagMax Pathogen RNA/DNA kit

RNA extraction was performed following the manufacturer's protocol. Each fecal sample (0.5 g) was added to a 2 mL tube containing 1 µL of PBS, vortexed vigorously to achieve complete suspension, and centrifuged at 100 × g for 30 seconds. For each reaction, 500 µL of lysis/binding solution was mixed with 2 µL of carrier RNA, while a separate mix of 10 µL nucleic acid binding beads and 10 µL lysis enhancer was prepared by vortexing. In a deep well plate, 500 µL of the lysis/binding mixture was dispensed into each designated well, followed by the addition of 200 µL of the fecal suspension. Plates were sealed and placed on a vigorous shaker for 5 minutes, then centrifuged at 2500 × g for 5 minutes to clarify the lysate. Wash plates were prepared by adding 300 µL of Wash Solution 1 to two 96-deep well plates and 450 µL of Wash Solution 2 to another two. An elution plate was prepared by dispensing 90 µL of elution buffer into each well of a standard 96-well plate. For sample processing, 20 µL of the bead/lysis enhancer mix was added to each well of a 96-deep well plate, followed by 400 µL of clarified lysate and 350 µL of 100% isopropanol, for a total volume of 750 µL per well. Sample, wash, and elution plates were then placed onto the extraction processor as directed. Final RNA elutions were stored at –20 °C for up to one month or at –80 °C for long-term preservation.

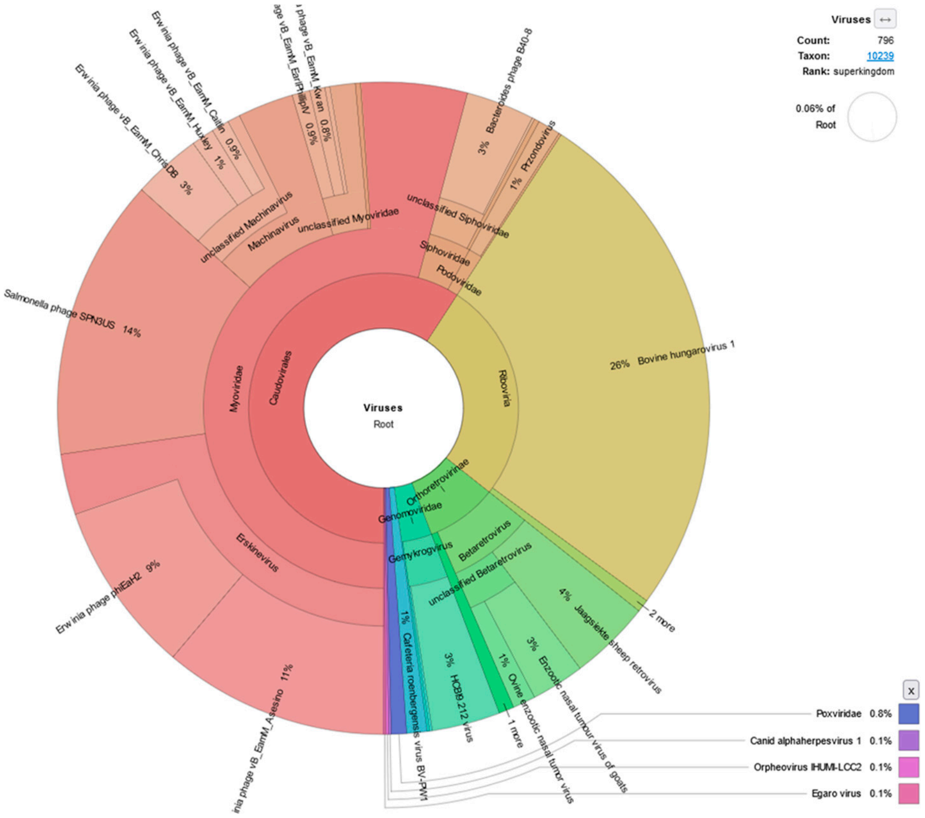

Supplementary Figure S1. Kraken Taxonomical Classification Analysis of raw FASTQ files for the caprine index case feces IL43480 showing 26% of reads classified as related to bovine hungarovirus 1 (in yellow).
